# 

**Published:** 1861-01

**Authors:** 


### CHICAGO MEDICAL JOURNAL.

### INDEX TO VOL. IV.

PAGE.

Alcoholic Spirits, Adulteration............ 51
Arsenic—Grease an Antidote............. 54
Arteria Innom—Ligature............. 54, 240
American Medical Association..... 55, 115, 123
186, 247, 250, 283, 289, 294
Alcohol and Tuberculosis........ 118, 269, 557
Ascarides .............................. 274
Alkalies—Action on Urine............... 280
Asphyxia ......................... 288, 725
Amputation—Brown ...................... 328
Atmospheric Corpuscles................. 401
Asthma, Palli. Treatment................ 422
Antaphrodisiac.......................... 543
Abdomen—Spont. Rupture............... 543
Anæsthetics—New ...................... 613
Alcohol in Disease....................... 639
Ammonia, Hydro Chlor................... 686
Asthma—Arsenic in ..................... 712
Ankle-Joint—Pirogoff's Operation........ 746
Army Rations............................ 751

Bloodletting *et alii*................. 238, 289
Bismuth, Action of....................... 295
Bedsores ................................ 368
Brigade Case............................ 517

Constipation—Batford.................... 15
Cold, Death by—Morbid Appearance...... 43
Cancer, Palliative Treatment ............ 53
Climate of California in Phthisis....... 53, 103
Cerium, Oxalate of—Jones............... 65
Croup, Ice Water in Treatment of......... 101
Cutaneous Discolorations—Laycock....... 136
Cornea, Foreign Bodies on—Holmes....... 177
Chicago, Mortality Statistics of....... 192, 291
Club Foot Dressings—Gutta Percha—Ray.. 193
Coagula in Circulatory System—Holmes... 207
Cimicifuga in Epilepsy and Chorea......... 292
Coffee in Pertussis ...................... 369
Cutaneous Diseases...................... 372
Cathartics, Use of........................ 413
Climate, Influence of, on Northern and Southern Constitutions .............. 455
Chancre, Primary from Secondary—Jones. 496
Cowards—A Contrast..................... 495
Chemical Therapeutics................... 519
Cadets, Medical.......................... 546
Cerebral Symptoms, etc.................. 601
Congestion of the Brain.................. 718
Clinical Teaching ........................ 738

Diabetes Mellitus......................... 80
Diphtheria, Treatment of—Thayer ....... 87
" " Local—Kneeland ... 97
" " —Jacobi........... 99
" —J. J. Morgan, 121, 241, 333
Dissection Wounds....................... 287
Dysentery.......................... 355, 471
Dilatation of Cervixuteri................. 356
Dentition and its Derangements........... 622
Diminutive Products of Labor............. 717
Death from Chloroform, Mode ............ 720
Diarrhœa, Camp......................... 723

PAGE.

Enteric Fever—Ewing..................... 81
Ethics, 117, (Pious Dodge) 283, 357, 369, 376, 493, 685
Ergot, Natural History and Therap. Uses.. 133
Ether—Bost. Com................... 217, 681
Examining Board............... 286, 493, 686
Erysipelas—Whitmire .................... 319
En Rapport............................. 268
Epilepsy from Ascarides .................. 530
Eye, Enucleation of—Holmes ............. 691
Errhine for Coryza....................... 722

Francis, J. W.—Obituary.................. 115
Fractures, Ununited—McDowell........... 119
Fluid Extracts........................... 187
Fœtus, Retention of Dead—Tripp......... 215
Fracture, Ununited—Brainard............. 218
Finger Nail Signs........................ 228
Fractures, Ununited—Store............... 259
" Swinburne's Method....... 283, 680
Fistula in Ano .......................... 296
Fracture Couch.......................... 533
Formulæ, Convenient..................... 669

Gonorrhœa ........................ 52, 129
Gunshot Wounds of Lung—Clapp......... 73
Glycosuria in Marsh Fevers............... 135
Galactics ............................... 229
Gelsemin in Spermatorrhœa .............. 234
Goitre Exopthalmic—Hamill.............. 338
Gunshot Wounds—Hœmorrhage........... 393
" in Loading Artillery.......... 457

Hæmaturia, Miasmatic—Low .............. 181
Hypochond, Insanity—General Paralysis.. 139
Hernia, Irreduc. Scrotal, Treatment—Russ, 214
Hydroeephalus, Puncture, Death.......... 221
Hypodermic Injections.................... 238
Hæmorrhoids...................... 249, 717
Homœopathy, Renunciation............... 546

Iron, Persulphate of.............. 51, 54, 294
Infibulation ............................. 235
Influenza ............................... 236
Iodine Solutions......................... 237
Infirmary, Eye and Ear, Chicago ........ 362
Ice internally in Uterine Hæmorrhage..... 380
Insanity—Definition...................... 467
" Puerperal...................... 468
Introductory, R. M. C.—Miller............ 563
Insanity, Moral........................... 726

Joints—Free Opening of Suppurating. .... 251
Journals, Medical—Farewell to....... 381, 719
Joint, Hip—Exsection..................... 736

Lead—Slow Poison, Inf. on Conception.... 227
Library, City Medical..................... 243
Lamp Bath............................... 441
Local Editors on Trust.................... 589
Lead in Phthisis.......................... 672
Labia Pudenda, Inflammation—Hoag...... 697

PAGE.
Muscular Poisons .......................... 34
Mercurials—Rationale of Action............ 275
Malpractice—Judge's Charge............. 315
Measles .................................. 355
Mamma, Inflammation.............. 374, 722
Midwifery—Certain Diseases.............. 423
Milk Sickness....................... 431, 438
Military Surgery—Guthrie............... 450
" Surgeons, Perils of ............. 547
Michigan Army Medical Appointments..... 687
Mercurials in Remittent................. 711

Necrosis of Tibia—Eaton. ............... 331
Nervous System.......................... 544
Narcotics—Notes on...................... 617

Obituary Resolutions—Mrs. De L. Miller.. 126
" Dr. Fountain.................... 244
" D. M. Reese..................... 308
Osteotomy, Subcutaneous—Brainard....... 297
Obstetric Cases—Jones................... 332
Ophthalmia, Purulent, of Armies—Holmes. 443
Opium and Quinia, Antag. Effects.... .... 448
Organization, Molecular Theory........... 629
Overwork, Penalty of. .................. 747

Physiology and Scepticism................ 144
Patients, Interrogation of................ 224
Phthisis, Iodide of Iron in............... 225
Potash, Chlorate of.......... ....234, 244, 496
Parotid, Extirpation of................... 250
Parotid Tumor, Removal—Ray............ 260
Practical Suggestions to Writers.......... 268
Puerperal Convulsions.......274, 365, 713, 717
Pessaries ................................ 287
Prurigo Pudeudi.......................... 373
Philantrophy ............................ 392
Pus Corpuscles in the Air................ 397
Pott's Disease....... ................... 541
Placenta Prævia—Brooks................. 586
Pregnancy—Interesting case.............. 696
Phthisis, Iodide of Iron in............... 710
" Contagious... .................. 721

Quinine as a Prophylactic.............371, 682

Resection of Knee-Joint—Freer.......... 1
Rush Med. College....................50, 492
" " " Graduates, 1861........ 191
" " " Museum ............... 242
" " " Alumni Meeting........ 285
Rheumatism, Bandages in................. 238
Ring Worm, True.......... ............ 314
Right Man for the Right Place.........364, 409
Rheumatism, Chronic..................... 722

Stimulation *vs.* Depletion................ 18
Scrofulous Lymphatic Glands, Treatment.. 38
Sydenham, New Soc. Publications......... 110
Snakebites—McDowell.................... 119
Stimulants............................... 120
Surgical Cases—Ammerman............. 202
She-Doctors.............................. 237
Stethescope.... ........................ 241
Spina Bifida—Anderson.................. 257
Syphilization. ......................... 272
Sydenham, &c........ .................. 281
Sugar Antidotal to Alcohol. ............ 287
Similia Similibus Curantur—Parks......... 382
St. Paul Academy of Med................. 361
Sanitary Commission.................... 371
Spina Bifida....................392, 394, 597
Sanitary Report, Extract................. 406
" Condition of Troops.........421, 510

Shoulder Straps......................494, 548
Surgical Principles, New.................. 616

Tongue, Altera. in Disease.............. 311
Typhoid Fever................... ....375, 549
Turpentine as an Anæsthetic............... 378
Tooth-Drawing without Pain.............. 378
Tetanus, Treatment........ ..........441, 544
Therapeutic Value of Sulph. Magnes., Oil Turpentine and Calomel... .......... 501

Uræmia ........................... .... 396

Uterine Disease.................524, 660, 748

Vacuum, Tendency to, in Cerebro Spinal Cavity—Brainard.................. 4
Valedictory Rush Med College—Allen..... 147
Vaccination, Protective Power—Noble..... 173
Variola, Pitting in....................... 357
Virchow's Pathology Views............... 472

Womb, Inversion of—Hamill .......... ... 167
" Retroversion, Instrument—Jones ...............................199, 252

Zinc, Oxide of, Uses..................... 543

### PROCEEDINGS OF SOCIETIES.

Chicago Academy..... ............. 45, 113
Aurora City............................. 45
Rock River Union............... ........ 47
Æsculapian, Paris, Ill................... 107
Hancock Co.............................. 184
Will Co............. . ................ 185
Champaign Co........................... 215
Illinois State........................... 217
DeWitt Co..................... 264, 348, 560
Connecticut State........................ 376

### BOOK AND PAMPHLET NOTICES.

Manual of Auscultation, etc—Barth & Roger —Pottenger ... ..................... 56
Handbook of Hospital Practice—Lyons.... 57
The True Physician—Watson............. 57
Malformations of Rectum—Bodenhamer... 61
Diseases of Women—Hodge.............. 62
Miasmatic Fever—Logan ............... 230
Movement Cure—Taylor................. 230
Diphtheria—Slade.... .................. 231
Treatise on Fever—Lyons .............. 231
Institutes of Medicine—Paine........... 231
Epitome of Braithwaite—Wells........... 252
Prac. Treat. on Phthisis Pulmon.—Lawson, 278
American Medical Biography—Gross...... 294
Handbook of Military Surgeon—Tripler & Blackman.............................. 308
Hamilton's Military Surgery.......... 310, 555
Physiology—Dalton...... ............... 349
Placenta, etc., Modus Operandi of Heat and Cold, etc.—O'Reilly.................. 358
Practice of Medicine—Maxson........... 365
Brown Sequard—Physiological, etc........ 499
Venereal Diseases—Burmstead............ 676
Uræmia—Morland......................... 678
Obstetrics—Bedford................... .. 700
Placenta Prævia, Read................... 705
Medical Jurisprudence—Taylor........... 708
Diseases of Women—West................ 716
Diseases of the Joints—Barwell........... 723

### RECEIPTS NOT HERETOFORE ACKNOWLEDGED.

| | |
|---|---|
| Dr Leitch, Wis., ½ of vol. 2, 3 and 4...... | $5 00 |
| " E Stewart, Mich , vol. 3........ .... | 2 00 |
| " J S Pashler, Ills., vol. 3.............. | 2 00 |
| " O Clark, Wis., vol. 3................... | 1 00 |
| " W M Avery, Mich., ½ of vol. 4......... | 6 00 |
| " J C Batdorf, Iowa, vol. 1............... | 2 00 |
| " D Rogers, Iowa, vol. 3.................. | 2 00 |
| " T Bevan, Ill., vol. 3.... . .......... | 2 00 |
| " Freer, Ill., adv......................... | 5 00 |
| " R F Baker, Ill , vol. 4.................. | 2 00 |
| " G W Carpenter, Iowa, vols. 2 and 3.. | 4 00 |
| " J R Pearce, Ill., vol. 3................. | 2 00 |
| " Van Nus, Iowa, vol. 4.................. | 2 00 |
| " G Vasey, Ill., vol 3.......... ...... | 2 00 |
| " C M Richmond, Iowa, vol. 3........... | 2 00 |
| " Geo. Egbert, Iowa, vol. 3............ | 1 00 |
| " Hutchinson, Iowa, vol. 3............... | 2 00 |
| " J H Robinson Iowa, vol. 3............ | 2 00 |
| " G V Ewing, Ill., vol. 4................ | 2 00 |
| " H J Vanwinkle, vol. 3......... ...... | 2 00 |
| " T Wilkins, Ill., ½ of vol. 3............ | 1 00 |
| " G C Holmes, Ill., ½ of vol. 3.......... | 2 00 |
| Dr T A Hill, Ill., 3....................... | 2 00 |
| " Bray, Ill., vol. 3....................... | 2 00 |
| " O Peabody, Iowa, vol. 4............... | 2 00 |
| " G W Hartman, Ill., vol. 4............. | 2 00 |
| " R W Hall, Iowa, vol. 4................ | 2 00 |
| " H Duncan, Ind., vol. 3................ | 2 00 |
| " J A Adrain, Iowa, vol 3............... | 6 00 |
| " L D Smedley, Ill., vol. 4.............. | 2 00 |
| " Ehrhardt, Ill., vol 3.................. | 2 00 |
| " J S Bullock, Ill., vol. 3............... | 3 00 |
| " H Steele, Ill., vol. 4.................. | 2 00 |
| " J H Griffith, Iowa, vol. 4.. ......... | 2 00 |
| " J W McKinney, Ill., vol. 4.... ...... | 2 00 |
| " A L Kinber, Ill., vol. 4................ | 2 00 |
| " N Zeal, Iowa, vol. 4.................. | 2 00 |
| " M Stahl, Iowa, vol. 3................. | 2 00 |
| " A Wiltsie, Iowa, vol. 4................ | 2 00 |
| " D Ray, Cal., vol. 4.................... | 2 00 |
| " A Carpenter, Iowa, vol. 4............ | 2 00 |
| " D W Stormont, Ill., vol. 4............ | 2 00 |
| " A Berchelmann, Ill., vol. 4........... | 2 00 |

